# A multiple imputation method based on weighted quantile regression models for longitudinal censored biomarker data with missing values at early visits

**DOI:** 10.1186/s12874-017-0463-9

**Published:** 2018-01-11

**Authors:** MinJae Lee, Mohammad H. Rahbar, Matthew Brown, Lianne Gensler, Michael Weisman, Laura Diekman, John D. Reveille

**Affiliations:** 1 Division of Clinical and Translational Sciences, Department of Internal Medicine, McGovern Medical School, The University of Texas Health Science Center at Houston, Houston, Texas USA; 2 Department of Epidemiology, Human Genetics, and Environmental Sciences, School of Public Health, The University of Texas Health Science Center at Houston, Houston, Texas USA; 3 Queensland University of Technology, Brisbane, Australia; 4 University of California, San Francisco, California USA; 5 Cedars-Sinai Medical Center in Los Angeles, Los Angeles, California USA; 6 Division of Rheumatology, Department of Internal Medicine, McGovern Medical School, The University of Texas Health Science Center at Houston, Houston, Texas USA

**Keywords:** Limit of detection, Left-censoring, Missing early visits, Quantile regression, Multiple imputation

## Abstract

**Background:**

In patient-based studies, biomarker data are often subject to left censoring due to the detection limits, or to incomplete sample or data collection. In the context of longitudinal regression analysis, inappropriate handling of these issues could lead to biased parameter estimates. We developed a specific multiple imputation (MI) strategy based on weighted censored quantile regression (CQR) that not only accounts for censoring, but also missing data at early visits when longitudinal biomarker data are modeled as a covariate.

**Methods:**

We assessed through simulation studies the performances of developed imputation approach by considering various scenarios of covariance structures of longitudinal data and levels of censoring. We also illustrated the application of the proposed method to the Prospective Study of Outcomes in Ankylosing spondylitis (AS) (PSOAS) data to address the issues of censored or missing C-reactive protein (CRP) level at early visits for a group of patients.

**Results:**

Our findings from simulation studies indicated that the proposed method performs better than other MI methods by having a higher relative efficiency. We also found that our approach is not sensitive to the choice of covariance structure as compared to other methods that assume normality of biomarker data. The analysis results of PSOAS data from the imputed CRP levels based on our method suggested that higher CRP is significantly associated with radiographic damage, while those from other methods did not result in a significant association.

**Conclusion:**

The MI based on weighted CQR offers a more valid statistical approach to evaluate a biomarker of disease in the presence of both issues with censoring and missing data in early visits.

## Background

With advances in biotechnology, biological markers (biomarkers) continue to play an important role in an increasing number of biomedical studies. Biomarkers have led to a better understanding of the natural history and development of acute and chronic diseases, providing insights of the mechanism of treatment effects to identify and classify patients into different risk categories, and potential biological pathways that can be used to guide the therapeutic strategies for future treatment targets. Biomarker data are often measured over a period of time in biomedical studies to determine if the temporal changes differ between the patients who develop disease and those who do not. For example, C-reactive protein (CRP) is one of the primary biomarkers known to reflect the degree of inflammation in the body and it has been widely used for studies of Ankylosing spondylitis (AS) to monitor disease activity, assess response to treatment and predict radiographic progression. However, in a longitudinal study, biomarker data may not be collected in certain time points for some patients. Furthermore, the biomarker data may be subject to censoring due to limits of detection (LOD). For example, in the Prospective Study of Outcomes in Ankylosing Spondylitis (PSOAS) [7], CRP data not only were censored due to limits of detection but also were incompletely collected at early study visits because blood sample collection was not a part of the original study design.

Compared to single imputation, it has been shown that model-based imputation techniques such as multiple imputation (MI) methods can account for the uncertainty about the prediction of the unknown missing values [21] and provide more valid statistical inference (Lubin [15]). Likelihood-based MI approaches have been proposed to address censoring issues due to limits of detection when the biomarker data are considered as covariates in a model. For example, Lee et al. [8] proposed MI for the multiple-censored correlated covariates based on the Gibbs sampling method. However, these methods focus on estimation of mean of biomarkers and assume normality of the distribution of biomarker data, which may not be valid as most biomarker data are highly skewed even after log-transformation. These limitations prompted development of alternative methods for non-normal biomarker data. For example, Powell [19, 20] proposed using quantile regression models for censored data (i.e., censored quantile regression) with detection limit and it has been extended for longitudinal data using improved computational methods (Wang and Fygenson [27]; Lee and Kong [9]; Sun et al. [23]). As an important alternative to the mean regression models, quantile regression models are increasingly used in longitudinal study due to its robustness to non-normality or heteroscedasticity and minimal assumption imposed on the quantiles of data. Estimating different quantiles should be of more practical interest especially in the presence of censoring issue, providing a broader picture of data distribution; specifically, it is common that some quantiles of biomarker data show significant effects that are not significant in other quantile. MI approaches that are based on censored quantile regression to handle censored covariates have been also proposed. For example, Wang and Feng [28] developed a multiple imputation approach for M-regression models. However, these approaches cannot handle multiple imputation in the presence of both censored and missing covariates in longitudinal data setting.

Lee and Kong [10] proposed an estimation approach based on censored quantile regression using the inverse probability weighting technique to handle longitudinal response variable with both censoring and monotone missingness [10] which is mainly caused by dropout; the basic concept of this method is that an individual’s contribution to the estimating equations is incorporated by the inverse probability weights for dealing with missing data at a dropout time. Since in PSOAS, CRP data for some patients were not completely collected at early study visits due to study design, this necessitated development of a new approach to handle missing data while controlling for censoring issue simultaneously.

In this paper, we propose a specific multiple imputation strategy that not only account for censoring, but also missing data at early visits when longitudinal biomarker data are modeled as a covariate. Assuming monotone missing pattern holds, we adopt the idea of Lee and Kong’s estimation method to establish weighted censored quantile models which are incorporated into our developed multiple imputation process. The focus here is assessing through simulation studies the performances of our multiple imputation approach by comparing relative efficiency of our method with that of complete case analysis and other traditional multiple imputation methods. We also illustrate application of the method to real life data from PSOAS to achieve realistic situations while specifically evaluating the association between CRP and radiographic damage.

## Methods

### The prospective study of outcomes in Ankylosing Spondylitis (PSOAS)

Ankylosing spondylitis is a chronic inflammatory disease characterized by inflammatory spinal pain usually beginning in the second to fourth decades of life that can result in chronic pain, that can result in functional impairment and diminished quality of life, and, in some patients, complete spinal fusion. In PSOAS, participants meeting the modified New York (mNY) Classification Criteria for AS [26] were enrolled from one of the five study sites (Cedars-Sinai Medical Center in Los Angeles, California, the University of Texas Health Science Center at Houston (UTH), the NIH Clinical Center, the University of California at San Francisco (UCSF), and the Princess Alexandra Hospital in Brisbane, Australia (PAH)^1^ and were followed for up to 13 years (through two cycles of NIH funding: 2002–2006 and 2007–2016). At each study visit, spaced 6 months apart, the patients underwent comprehensive clinical evaluation for disease activity and functional impairment. Self-reported outcomes were measured at 6-month intervals and radiographic data, including AP pelvis X-rays, AP and lateral lumbsacral spine films and lateral cervical spinal films were collected every 2 years in order to assess longitudinal radiographic damage which was defined by scoring the Bath Ankylosing Spondylitis Radiology Index (BASRI) [17] and the modified Stoke Ankylosing Spondylitis Spine Score (mSASSS) [3]. C-reactive protein (CRP) levels and erythrocyte sedimentation rate (ESR) as well as medication usage were also determined at each clinical visit.

### Analysis cohort

**Fig. 1:**
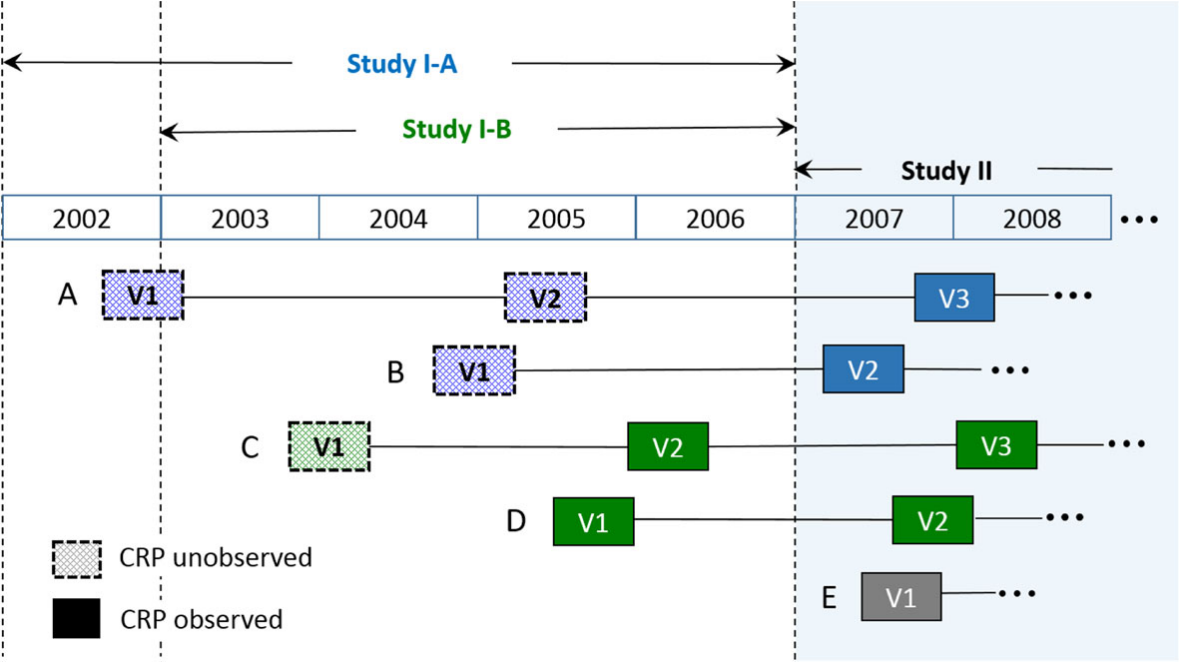
Patients’ X-ray visits (organized at ≥ 2-year intervals) over time with indication of unobserved and observed CRP data. Patients in the cohort comprises three different groups of patients depending on the study that they were first enrolled in: **Study I-A** CRP data collection was not a part of the protocol (Patient A and B); **Study I-B** The original protocol had a screening visit where X-rays were collected but no blood samples (Patient C), but a protocol amendment led to a combining of the screening and baseline visit resulting in blood and X-rays collected at the first visit (Patient D); **Study II** both blood samples and X-rays were collected starting from their first visit (Patient E)

One of the objectives of PSOAS was to evaluate factors associated with longitudinal radiographic severity and rate of progression in AS patients. Specifically, we focused on evaluating longitudinal association between CRP level and radiographic damage which is assessed by mSASSS values (range 0–72) at each X-ray visit. We considered analysis cohort of 295 patients who were confirmed AS by mNY criteria and had at least 4 years of radiologic follow up data (as of August 2016). However, we faced with two challenges in analyzing CRP data in relation to mSASSS. First, we found that 13.3% of CRP values were left-censored due to being below the limit of detection. Another issue is related to unobserved CRP data during early visits for some patients which was by study design. Specifically, of the 295 patients with at least 4 years of radiologic follow up, 37% have been followed since first cycle of funding, i.e., Study I (enrolled from 2002–2006)^2^ and then consented to Study II (enrolled from 2007–2016) for their continued participation. The inclusion of these patients increases the study power by increasing the number of patients who have been followed for 10+ years. However, CRP levels for 111 patients among our study cohort were not collected for up to first two consecutive X-ray visits because initially blood sample collection (e.g., CRP and ESR) was not a part of the protocol for some patients in Study I. Different scenarios in which missing CRP data are generated are presented with detailed descriptions in Fig. 1. An important feature of CRP data was that it has a monotonic missing pattern; i.e., if a value was missing at visit t then the values for all previous visits (i.e., *k*,1≤*k*≤*t*−1) were also missing. This monotonic pattern was found in 92% of 111 patients. We also discovered that the number of patients who had CRP data missing at their first visit only (73.9%) was higher than the number of those who had missing data for both first and second visits (26.1%). There were only 5 patients who had CRP levels missing at all first three visits. Also we noted that patients who were enrolled earlier had a higher number of visits with unobserved CRP data.

### Statistical approach

Our approach for assessing the longitudinal association between CRP and radiographic damage (i.e., mSASSS) includes four steps: Step (1) modeling missing data processes, Step (2) applying the inverse weighting techniques to censored quantile regression (CQR) using the probabilities of missing early visits that are estimated from the modeled missing data process, Step (3) employing multiple imputation process for both censored and missing CRP data at early visits, based on quantile estimates from established weighted CQR in Step (2), and Step (4) conducting longitudinal regression analyses where imputed CRP data are treated as an independent variable and mSASSS as a response variable. Natural log-transformed CPR was used in our analyses to reduce its highly right-skewed distributions.

Step (1) Since the true probability of missing data is unknown, we modeled missing data process for each visit separately, through binary logistic regression with a response variable which indicates whether CRP data are observed or not (i.e., 1 = observed; 0 = missing) and independent variables that include observed CRP data at later times (i.e., the first available CRP) and other variables that were associated with missingness of CRP. Using the predicted probabilities (=*η*_*it*_ for the *i*-th patient and the *t*-th visit) that were estimated from these models, we derived the patient-level probability weights (=*π*_*i*_) under monotone missing data mechanism. Details regarding derivation of probability weights are shown in Appendix A.2. Although other reasons may lead to informative missing, we predicted the probability of missing CRP at early visits conditional on the observed CRP data at the rest of follow up visits (if censored, imputed by half of detection limit (DL), i.e., DL/2), and a set of covariates including study sites and the study group that patients were first enrolled in.
Step (2) Censored quantile estimating equation incorporated by inverse probability weights was defined as a function of the variables that were significantly associated with CRP. Details regarding specific models and estimation procedure are presented in Appendices A.1 and A.2. In practice, it is important to use all available information to build the best imputation model [18, 21, 22]; we conducted the weighted CQR model based on the variables that include the covariates and the outcome of the potential analysis models even if they have limited predictive power. Once weighted CQR model was established, parameter estimates of different quantile levels were obtained by implementing function ‘crq’ in the R package quantreg for the existing optimization algorithms [4, 21]. Specifically, we used the option ‘Powell’ for method and ‘left’ for censoring type (i.e., ctype). It is well known that even if the missing data depend on the observed data, the weighted estimating equations provide unbiased estimation, when the missing data process is modeled with correctly specified probability [10, 12].
Step (3) Missing CRP data were imputed by the *u*-th conditional quantiles based on quantile-specific parameter estimates of aforementioned weighted CQR, where random variable U was generated from a uniform distribution between 0 and 1. We can estimate quantile-specific parameters using R function ‘crq’ with a function argument called ‘tau’, the quantile level at which the model is to be estimated. For censored CRP data, we first estimated the conditional probability of censoring, denoted by *ω*, using longitudinal logistic regression model with adjustment of potential predictors of censoring, such as study sites (because censoring rates varied over study sites from 2% to 29%), ESR levels, functional outcomes [2], disease activity [6] and medications usage. Then we used *ω* to randomly generate values of v from a uniform distribution between 0 and *ω*, which were used to impute the censored value by the *v*-th conditional quantile. Since the conditional probability of censoring was estimated from logistic regression model and the imputations are obtained from a separated CQR, in few samples, the imputed values may not be less than a desired detection limit. When this situation occurs, we discarded the corresponding cases and used another *v* value, sampled from a uniform distribution between 0 and *ω*.
Step (4) After Step (3) was repeated *M*=5 times to generate five imputed datasets, we conducted longitudinal regression analyses to evaluate association between CRP and dependent variable, mSASSS for each of imputed datasets. Details of analysis model are described in “Analysis of PSOAS data” section. To obtain the parameter estimates of interest, we defined the combined MI estimator as a mean of five estimates. Variance of MI estimators was determined based on 500 bootstrap samples, by resampling the observations with replacement and *p*-values were calculated assuming the normality of estimated parameters. Additional details related to our imputation procedures are discussed in Appendix A.3.

### Simulation studies

We conducted simulation studies to investigate the performance of our developed MI methods through different scenarios. We considered the following four different scenarios of longitudinal data structures, as well as different levels of censoring for generating biomarker data.

1. Multivariate normal (MVN) distribution ; exchangeable covariance structure
2. Multivariate normal (MVN) distribution ; unstructured covariance
3. Multivariate exponential (MVE) distribution; exchangeable covariance
4. Multivariate exponential (MVE) distribution; heteroscedastic covariance structure (i.e., covariance depends on a set of covariates)

In order to generate a longitudinal outcome variable that mimics the distribution of mSASSS (=*y*_*it*_) in PSOAS, we used the following regression model

1 $$\begin{array}{@{}rcl@{}} y_{it}=\alpha_{0} + \alpha_{1} z^{*}_{it}+ \alpha_{2} w_{it} +\epsilon_{it},  \end{array} $$

for the *i*-th patient and the *t*-th visit, where *α*_0_=5, *α*_1_=−4, *α*_2_=−6, *w*_*it*_ represents the longitudinal structure variable time (*t*=1, ⋯,4) and $z^{*}_{it}$ denotes complete biomarker data which have been generated based on the aforementioned four scenarios. An error term *ε*_*it*_ was generated from multivariable normal distribution based on exchangeable covariance structure with a correlation coefficient *ρ* of 0.3. We then produced missing and censored values for biomarker data *z*_*it*_ that mimic the missing data pattern of CRP levels in PSOAS. We used a logistic regression to model probability of observed biomarker data *η*_*it*_, based on variables *w*_*it*_, *y*_*it*_ and the first observed biomarker data after time *t* (i.e., $z_{it'}, t<t'\leq 4\phantom {\dot {i}\!}$). Based on probability *η*_*it*_, we calculated the patient-level probability weights (=*π*_*i*_) under monotone missing data mechanism through a specific function of *η*_*ij*_, as shown in Appendix B. For generating censored data, we chose the detection limit c, as the (100×*r*)-th percentile of the simulated biomarker data, where r is the censoring rate (i.e., *r*=0.1, 0.15, 0.2, 0.3). We simulated data for 75% of patients who had missing data for up to first 3 visits (i.e., missing at first visit only, first and second visits, or all first three visits), and 25% of patients who had complete measurements up to visit 4. For each scenario, three hundred simulation datasets with sample size of 250 were generated. Details regarding parameters used for data generation and covariance structures for each scenario are described in Appendix B.

For multiple imputation, we fitted weighted CQR (wCQR) models using both known probability weights *π* (MI-wCQR _1_) and $\hat {\pi }$ that was estimated through the aforementioned logistic regression model. Since the observed biomarker data *z*_*i*,*t*+*j*_ in this logistic regression model had also censored values, we considered fitting two separate wCQR models, one based on imputed censored data by DL/2 (MI-wCQR _2_) and the other using uncensored data only (MI-wCQR _3_), in order to see the impact of these two approaches on parameter estimates. We also considered unweighted CQR method (MI-CQR) which accounts for censoring but ignores the missing data mechanism. Other imputation methods were further applied, that included Markov Chain Monte Carlo (MCMC)-based MI methods [11, 14] through on Bayesian frame work for a monotone missing data, which were implemented in the function ‘mice’ of the R package mice [25], imputing only missing values (MI-MCMC _1_), as well as imputing both censored and missing values (MI-MCMC _2_). MCMC-MI algorithm obtains the posterior distribution of parameters by sampling iteratively from conditional distributions based on Gibbs sampling method.

Using imputed biomarker data generated from different MI methods described above, we conducted longitudinal regression analysis using model 1, for each scenario. For assessing the performance of each estimator, we calculated bias and ratio of the mean squared error (MSE) of the omniscient estimator (OMNI), the gold standard, which is based on the complete data without censoring, to that of each estimator. Throughout we refer to this ratio of MSEs as relative efficiency (RE), which will be used for comparing the performance of the aforementioned methods. Moreover, we also conducted complete case analysis using only observed biomarker data where censored data were imputed by a single value of DL/2 (CC-DL/2).

### Analysis of PSOAS data

Censored or missing CRP data at early visits in PSOAS were imputed based on six different approaches (CC-DL/2, MI-MCMC _1_, MI-MCMC _2_, MI-CQR, MI-wCQR _2_, MI-wCQR _3_) that are described in “Simulation studies” section. For each imputed dataset, we assessed the longitudinal association between natural log-transformed CRP levels and mSASSS while controlling for potential confounding factors, that included Bath Ankylosing Spondylitis Disease Activity Index (BASDAI), medication usages of Tumor Necrosis Factors inhibitors (TNFi), and Nonsteroidal Anti-Inflammatory drugs (NSAIDs) as well as demographic information such as sex, race, disease duration, co-morbidity, education and smoking status. Multivariable mixed effect Poisson regression models were conducted for each imputed dataset, to account for the correlations of repeated measures within a patient.

## Results

### Simulation study results

Table 1 presents the results of simulation study across different censoring rates based on aforementioned Scenario 1; Bias and relative efficiency (100 × RE) are presented for each parameter, *α*_0_, *α*_1_, *α*_2_. Overall, our proposed three MI-wCQR approaches (i.e., MI-wCQR _1_, MI-wCQR _2_, MI-wCQR _3_) produced more efficient estimators (i.e., higher REs) than other MI methods that were used for comparison. Specifically, with 10% censored data, RE of our MI methods for *α*_1_, the coefficient of biomarker, ranged from 53.4% to 53.8%, while for CC-DL/2 RE was 3.2% and for that of MI-MCMC _1_, MI-MCMC _2_ and MI-CQR were 5.0, 39.5, and 49.6%, receptively. Although as the censoring rate increased the magnitude of RE for our methods decreased slightly, we still observed higher REs, ranging from 40.1% to 44.9% for *α*_1_, compared to other methods with REs ranging from 8% for CC-DL/2 to 39.98% for MI-CQR, when 30% of data were censored. We also observed similar patterns in REs for other two coefficients, *α*_0_ and *α*_2_.

**Table 1:** Simulation results (10-30% censored; Scenario 1)

*α*	*α*_0_		*α*_1_		*α*_2_	
Method	Bias	100xRE	Bias	100xRE	Bias	100xRE
*10% censored*						
OMNI	0.0015	–	-0.0004	–	-0.0009	–
CC-DL/2	-0.0353	40.885	0.2295	32.190	0.0163	49.142
MI _1_	0.0151	19.644	-0.1900	15.025	-0.0531	29.805
MI _2_	0.0289	54.944	0.0044	39.458	-0.0055	67.701
MI-CQR	0.0588	61.407	-0.0237	49.594	-0.0133	80.753
MI-wCQR _1_	0.0548	64.175	-0.0142	53.410	-0.0109	82.931
MI-wCQR _2_	0.0554	64.336	-0.0143	53.619	-0.0110	83.657
MI-wCQR _3_	0.0562	63.070	-0.0119	53.832	-0.0112	82.383
*15% censored*						
OMNI	0.0015	–	-0.0004	–	-0.0009	–
CC-DL/2	0.0155	34.977	0.1073	20.623	0.0005	43.807
MI _1_	0.2081	12.694	-0.2884	11.962	-0.0673	20.976
MI _2_	0.0318	51.749	0.0043	33.300	-0.0061	65.404
MI-CQR	0.0612	60.611	-0.0265	48.581	-0.0131	80.495
MI-wCQR _1_	0.0541	62.868	-0.0195	52.850	-0.0097	81.684
MI-wCQR _2_	0.0521	61.869	-0.0194	51.751	-0.0060	80.580
MI-wCQR _3_	0.0566	61.969	-0.0155	51.419	-0.0104	81.064
*20% censored*						
OMNI	0.0015	–	-0.0004	–	-0.0009	–
CC-DL/2	0.0769	25.633	-0.0500	14.061	-0.0084	35.731
MI _1_	0.2753	11.360	-0.3920	10.965	-0.0793	15.871
MI _2_	0.0339	47.699	0.0041	28.147	-0.0067	61.568
MI-CQR	0.0636	59.024	-0.0273	44.248	-0.0129	75.347
MI-wCQR _1_	0.0542	62.054	-0.0215	49.542	-0.0087	79.569
MI-wCQR _2_	0.0485	62.323	-0.0233	48.830	-0.0066	78.507
MI-wCQR _3_	0.0618	60.503	-0.0178	45.908	-0.0112	75.721
*30% censored*						
OMNI	0.0015	–	-0.0004	–	-0.0009	–
CC-DL/2	0.2094	12.909	-0.4255	7.810	0.0062	23.053
MI _1_	0.4420	8.714	-0.6175	10.370	-0.0973	10.664
MI _2_	0.0383	42.703	0.0019	21.804	-0.0076	54.866
MI-CQR	0.0621	58.587	-0.0198	39.975	-0.0111	75.797
MI-wCQR _1_	0.0466	61.367	-0.0177	44.947	-0.0042	77.979
MI-wCQR _2_	0.0327	62.075	-0.0262	43.389	0.0018	79.091
MI-wCQR _3_	0.0302	61.427	-0.0072	40.062	0.0015	75.897

OMNI: Omniscient; CC-DL/2: CC with censored values imputed by DL/2; MI-MCMC _1_: MI-MCMC imputing only missing values; MI-MCMC _2_: MI-MCMC imputing both censored and missing values; MI-CQR: MI-unweighted CQR; MI-wCQR _1_: MI-weighted CQR using original probability of missing; MI-wCQR _2_: MI-weighted CQR using estimated probability from censored values imputed by DL/2; MI-wCQR _3_MI-weighted CQR using estimated probability from uncensored values only; RE: Relative Efficiency

Similar findings were observed under Scenario 2-Scenario 4, as shown in Table 2. Our MI methods provided higher REs compared to the other methods across all these three scenarios in the presence of 30% censoring. It also demonstrates that our weighted CQR methods is not sensitive to the choice of covariance structure, as compared to MCMC-based methods that assume normality of biomarker data. For example, RE for MI-MCMC _2_ under Scenario 3 (MVE) was about 49% lower than that of Scenario 2 (MVN) (i.e., from 21.52 for Scenario 2 to 11.07 for Scenario 3), while our methods provided consistent REs (< 0.5% change) over all three scenarios.

**Table 2:** Simulation results (30% censored; Scenario 2–Scenario 4)

*α*	*α*_0_		*α*_1_		*α*_2_	
Method	Bias	100xRE	Bias	100xRE	Bias	100xRE
*Scenario 2: MVN, Unstructured covariance*						
OMNI	0.0017	–	-0.0006	–	-0.0013	–
CC-DL/2	0.0527	16.527	-0.5413	7.541	0.1161	28.516
MI _1_	0.1809	8.011	-0.3166	11.718	0.0028	18.080
MI _2_	0.0183	42.410	-0.0014	21.517	-0.0035	52.201
MI-CQR	0.0433	63.613	-0.0050	36.069	-0.0089	69.551
MI-wCQR _1_	0.0319	71.852	-0.0099	42.007	-0.0031	78.523
MI-wCQR _2_	0.0204	71.104	-0.0299	40.525	0.0031	77.691
MI-wCQR _3_	0.0172	63.936	-0.0168	37.197	0.0030	70.009
*Scenario 3: MVE, Exchangeable covariance*						
OMNI	0.0028	–	0.0000	–	-0.0014	–
CC-DL/2	0.2094	12.616	-0.4212	7.660	0.0057	22.869
MI _1_	0.4430	3.682	-0.6098	3.640	-0.0981	10.591
MI _2_	0.0423	32.878	0.0008	11.074	-0.0088	44.840
MI-CQR	0.0667	58.224	-0.0183	36.201	-0.0128	71.372
MI-wCQR _1_	0.0501	62.644	-0.0163	41.087	-0.0055	77.240
MI-wCQR _2_	0.0379	62.214	-0.0256	40.690	0.0000	77.128
MI-wCQR _3_	0.0273	59.215	-0.0041	37.352	0.0018	71.902
*Scenario 4: MVE, Heteroscedastic covariance*						
OMNI	0.0028	–	0.0000	–	-0.0014	–
CC-DL/2	0.1739	14.389	-0.4081	8.210	0.0171	23.356
MI _1_	0.1892	3.537	-0.3084	1.531	0.0002	10.432
MI _2_	0.0410	32.650	0.0011	11.025	-0.0083	44.126
MI-CQR	0.0649	58.870	-0.0141	36.739	-0.0125	71.618
MI-wCQR _1_	0.0492	63.492	-0.0121	41.733	-0.0054	78.362
MI-wCQR _2_	0.0340	63.222	-0.0223	40.015	0.0009	78.325
MI-wCQR _3_	0.0273	59.195	-0.0041	37.005	0.0018	71.929

OMNI: Omniscient; CC-DL/2: CC with censored values imputed by DL/2; MI-MCMC _1_: MI-MCMC imputing only missing values; MI-MCMC _2_: MI-MCMC imputing both censored and missing values; MI-CQR: MI-unweighted CQR; MI-wCQR _1_: MI-weighted CQR using original probability of missing; MI-wCQR _2_: MI-weighted CQR using estimated probability from censored values imputed by DL/2; MI-wCQR _3_MI-weighted CQR using estimated probability from uncensored values only; RE: Relative Efficiency

**Fig. 2:**
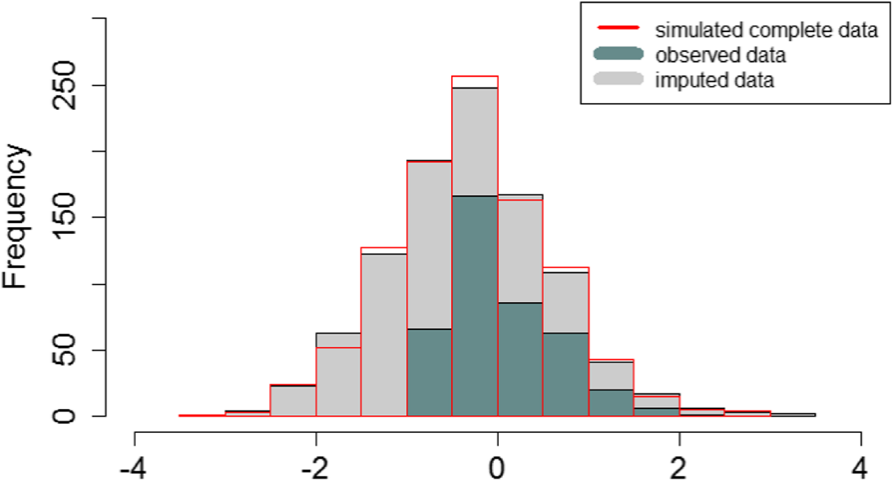
Imputed data distribution based on MI-wCQR _2_. Distribution of biomarker data from one of simulated datasets, distinguishing observed data (green area; data with left-censored/missing values) from imputed data (gray area); after imputation, data distribution was very similar to the complete data distribution that were originally simulated (red line)

Figure 2 displays distribution of biomarker data from one of simulated datasets, distinguishing the observed data from imputed data by our MI-wCQR _2_ method; the distribution of data after imputation was very similar to the complete data distribution that were originally simulated. Similar findings were observed for our other methods, MI-wCQR _1_ and MI-wCQR _3_ (Figures are not shown).

### Results of applying the proposed methods to PSOAS data

#### General characteristics of patients in PSOAS

Among 295 AS patients who had at least 4 years of radiologic follow up, the mean follow up time was 6.49 years (standard deviation (SD) = 2.37) with maximum years of 13.5 and mean number of X-ray visits was 3.6 (SD = 1.2). The cohort was 76.3% male, 81% white, 8.8% Hispanic, with a mean age 42.6 years (SD = 13.1) and a mean disease duration 18.0 years (SD = 12.7) at baseline. Of the 295 patients, 54 (18.3%) were from UCSF, 83 (28.1%) from UTH, 106 (35.9%) from Cedars Sinai, 42 (14.2%) from the NIH Clinical Center and 10 (3.4%) were from PAH. At baseline visit, 71.5% of patients had at least one comorbidity, 41.5% were ever-smokers and 10.7% were current smokers. A median mSASSS at baseline visit was 5 (inter-quartile range (IQR) = [0, 24]), the first observed CRP level with censored values imputed by DL/2 had a median of 0.4 (IQR = [0.2, 0.8]) and patients with mSASSS ≥ 4 had higher median CRP level compared to those with mSASSS < 4 (0.43 vs. 0.31).

#### Analysis results

Table 3 shows the adjusted rate ratios (RR) and *p*-values of complete case analysis using censored CRP data imputed by DL/2 (CC-DL/2), and those from imputed CRP levels by three other methods: MI-MCMC2, MI-CQR and MI-wCQR _2_. The results from MI-MCMC _2_ and MI-wCQR _2_ were very similar to those from MI-MCMC _1_ and MI-wCQR _3_ respectively (data not shown). This may be because the censoring rate of CRP in PSOAS data is not high enough to cause differences between these methods. However, there were noticeable differences in the estimates and the corresponding *p*-values for CRP across these four methods. The results from our method (MI-wCQR _2_) suggest that higher CRP is significantly associated with radiographic damage (adjusted RR = 1.018; 95% confidence interval (CI) = [1.004, 1.031]; *p*=0.0095), while the other methods did not result in a statistically significant association (*p*=0.99 for CC-DL/2; *p*=0.56 for MI-MCMC _2_; *p*=0.18 for MI-CQR).

**Table 3:** Analysis results of longitudinal association between CRP and mSASSS when CRP levels were imputed by different imputation methods

	log(CRP)	
Method	adj. RR (95% CI)	*p*-value
CC-DL/2	1.001 (0.98, 1.02)	0.9867
MI-MCMC _2_	1.006 (0.99, 1.03)	0.5586
MI-CQR	1.010 (0.995, 1.02)	0.1839
MI-wCQR _2_	1.018 (1.004, 1.03)	0.0095

CC-DL/2: CC with CRP imputed by DL/2; MI-MCMC _2_: MI-MCMC imputing both censored and missing CRP; MI-CQR: MI-unweighted CQR; MI-wCQR _2_: MI-weighted CQR using estimated probability from censored CRP imputed by DL/2; adj. RR: adjusted Rate Ratio after controlling for sex, race, disease duration, co-morbidity, education, smoking status, BASDAI and medication usages of TNFi and NSAIDs

## Discussion and conclusion

Biomarker data are often subject to left censoring due to inability to obtain complete data when the measurements are below the limit of detection. In longitudinal studies, it is also possible that biomarker data are not completely collected during early study visits which introduces a monotonic missing data pattern. Both likelihood based joint modeling techniques ([5, 16, 24]) and quantile regression approaches ([10, 12, 29]) have been used to deal with monotonic missing data. However, most of these methodological developments have dealt with monotone missing data caused by termination from a trial or study (e.g., dropout), to our knowledge there are no published studies that have developed imputation approaches that specifically accommodate both censoring and missing data at early follow up visits. In this article we have developed the use of multiple imputation procedure that is based on weighted censored quantile regression model to account for both left-censored and monotone missing biomarker data during early visits. Specifically, we applied inverse probability weighting techniques to incorporate missing data in early visits through a multiple imputation based on censored quantile regression.

Our findings from the simulation study indicate that our proposed method performs better than other MI methods as assessed by higher RE. Further, our approach is not sensitive to the choice of covariance structure as compared to other methods that assume normality of biomarker data. The results of our method MI-wCQR _2_, where missing data probability weights were estimated based on the imputed censored data by DL/2, were similar to those of MI-wCQR _1_, where the true probability weights were used. This is reassuring to use estimated probabilities for missing data based on the logistic regression model for missing data process, as in real life applications we may not have information about the true probabilities for missing data. However, when the uncensored data were only used for the missing data model (MI-wCQR _3_), the results were not as good as the ones from MI-wCQR _1_ and MI-wCQR _2_ (i.e., lower RE). Since missing data model is defined by linear covariate effects, uncensored data can be used to obtain a consistent estimate of probability of missing, but it may introduce some bias when there is a strong underlying nonlinear relationship or the censoring rate is considered high (e.g., > 30%). This is consistent with literature that indicate accurate estimates of probability is critical when the inverse weighted probability based approach is applied [27, 29].

Moreover, we demonstrated application of our methods to real data from the PSOAS cohort by examining the longitudinal association between CRP levels and radiographic damage in a situation where CRP levels for some patients were either not collected in the early visits or left-censored due to the detection limit. The results from our method indicated that higher CRP is significantly associated with radiographic damage, while the other methods did not result in a significant association. This finding is also consistent with clinical expectation that CRP is associated with radiographic severity in patients with AS [1].

Though developed method could be implemented with standard software package quantreg in R that fits quantile regression, we are currently developing R package for users to easily implement the proposed MI approaches. Censored quantile regression has been extended to data censored at both lower and upper thresholds [4], therefore our method can be also directly extended to doubly censored biomarker data. There is a growing interest in developing MI methods that impute missing data across multiple medications while accounting for the correlations among them, which can be also extended by our proposed method.

Based on our earlier work (Lee and Kong [10]), we expect our method to provide consistent estimates for the parameters in the weighted quantile regression model, assuming the missing data model is correctly specified [10, 12]. Despite aforementioned advantages for our method shown earlier, we acknowledge that the misspecification of the model for missing data process may introduce bias in the estimation of parameters. Therefore, it is important to identify the model carefully and interpret the analysis results cautiously [8, 10].

## Appendix A: Model formulation/ Estimation procedure

### A.1 Weighted censored quantile regression model accounting for missing early visits

Let $z^{*}_{it}$ be the biomarker measurement for the *i*-th subject at time *t* assuming all subjects are to be observed at the same time. Suppose we define the linear regression model $z^{*}_{it}= \boldsymbol x_{it}^{T}\boldsymbol \beta +e_{it},\quad i=1,\cdots, n; \quad t=1,\cdots, m \label {latent}$, where ***x***_*it*_ is a *p*×1 vector of covariates that can include the time of measurement, ***β*** is an unknown *p*×1 vector of regression parameters and the random errors *e*_*it*_ are correlated within the subject to reflect the serial correlations of repeated measurements within each individual. If the *τ*-th conditional quantile of *e*_*it*_ given $\boldsymbol x_{it}^{T}$ is assumed to be zero, a quantile regression model related to the *τ*-th quantile of response variable, $q_{\tau }\left (z^{*}_{it}\right)$, conditional on *x*_*it*_ has the form $q_{\tau }\left (z^{*}_{it}\right)=\boldsymbol x_{it}^{T}\boldsymbol \beta _{\tau }, \quad 0<\tau <1$, where ***β***_*τ*_ is a vector of quantile specific regression parameters corresponding to the coefficient ***β*** in the linear regression model above. When there exists a lower detection limit, say *c*, $z^{*}_{it}$ is a latent variable and we cannot observe the biomarker measurement if it has a value below *c* and we only observe $z_{it}=z^{*}_{it}$, if $z^{*}_{it}>c$. This leads to the longitudinal censored quantile regression (CQR) model defined as $z_{it}= max\left (c, \boldsymbol x_{it}^{T}\boldsymbol \beta +e_{it}\right)$. We can define the objective function for longitudinal censored data as

2 $$\begin{array}{@{}rcl@{}} Q_{n}(\boldsymbol\beta_{\tau})=\frac{1}{n}\sum\limits_{i=1}^{n}\sum\limits_{t=1}^{m}\rho_{\tau}\left(z_{it}-max\left\{c,\boldsymbol x_{it}^{T}\boldsymbol\beta_{\tau}\right\}\right).  \end{array} $$

The loss function *ρ*_*τ*_(*u*)=*u*{*τ*−*I*(*u*≤0)}, with *I*(·) being an indicator function, represents the contribution by residuals. The estimates resulting from (2) are equivalent to the solution of estimating equation

3 $$ { S_{n}(\boldsymbol\beta_{\tau})=\frac{1}{n}\sum\limits_{i=1}^{n}\sum\limits_{t=1}^{m}\boldsymbol x_{it}\left[\tau-I\left(z_{it}\le max\left\{c,\boldsymbol x_{it}^{T}\boldsymbol\beta_{\tau}\right\}\right)\right]=0.}  $$

To apply the weighting techniques to the censored quantile regression model for handling missing data at early visits, let *O*_*i*_ be a random variable indicating the time point when the data collection was started for the *i*-th subject. *O*_*i*_ can take the values between 1 and *m*. If the subject has completed 1 ∼*m* follow-up visits then *O*_*i*_=1, and if the subject had missing data from visit 1 to *m*-1 then *O*_*i*_=*m*. We denote $\boldsymbol z_{i}^{o}$ as the observed response history since the data collection was started, and ***X***_*i*_={***x***_*i*1_,⋯,***x***_*im*_}^*T*^ as a set of covariates that were observed from the complete study visits 1 ∼*m*. When the biomarker measurements are MAR, the conditional probability of missing early visit from the baseline to the *o*_*i*_−1 occasion for the *i*-th subject is $\pi _{io_{i}}=Pr\{O_{i}=o_{i}|\boldsymbol z_{i}^{o}$, ***X***_*i*_,***γ***} (*o*_*i*_=1,⋯,*m*), where $\pi _{io_{i}}>0$ and ***γ*** is a parameter vector of the regression model. Now the weighted estimating equations for censored quantile regression model can be defined as

4 $${} {{\begin{aligned} S^{w}_{n}(\boldsymbol\beta_{\tau})=& \left (\sum\limits_{i=1}^{n}\frac{1}{\pi_{io_{i}}}\sum\limits_{t=o_{i}}^{m }\boldsymbol x_{it}\left[\tau-I\left(z_{it}\le max\left\{c_{i},\boldsymbol x_{it}^{T}\boldsymbol \beta_{\tau}\right\}\right)\right] \right)\\ =&\sum\limits_{i=1}^{n}\left(\sum\limits_{o_{i}=1}^{m}\frac{I(O_{i}=o_{i})}{\pi_{ij}}\sum\limits_{t=o_{i}}^{m}\boldsymbol x_{it}\left[\tau-I\left(z_{it}\le max\left\{c_{i},\boldsymbol x_{it}^{T}\boldsymbol \beta_{\tau}\right\}\right)\right]\right) \end{aligned}}}  $$

The basic idea of weighted estimating equations is to weight each subject’s contribution by the inverse probability of missing early visits to a given occasion. After we define $\boldsymbol x_{it}^{w}=\frac {1}{\pi _{io_{i}}}\boldsymbol x_{it}, z_{it}^{w}=\frac {1}{\pi _{io_{i}}}z_{it}$ and $ {c_{i}}^{w}=\pi _{io_{i}}^{-1} {c}$, Eq. (4) can be written in the same form as the unweighted estimating Eq. (3) as follows:

$${} {{\begin{aligned} S^{w}_{n}(\boldsymbol\beta_{\tau})&=\frac{1}{n}\sum\limits_{i=1}^{n}\sum\limits_{t=o_{i}}^{m}\pi_{io_{i}}^{-1}\boldsymbol x_{it}\left[\tau-I\left(\pi_{id_{i}}^{-1}z_{it}\le max\left\{\pi_{io_{i}}^{-1} {c},\pi_{io_{i}}^{-1}\boldsymbol x_{it}^{T}\boldsymbol\beta_{\tau}\right\}\right)\right] \\ &=\frac{1}{n}\sum\limits_{i=1}^{n}\sum\limits_{t=o_{i}}^{m}\boldsymbol x_{it}^{w}\left[\tau-I\left(z_{it}^{w}\le max\left\{{c_{i}}^{w},\boldsymbol x_{it}^{w T}\boldsymbol\beta_{\tau}\right\}\right)\right]=0.  \end{aligned}}}  $$

Thus, the corresponding objective function is in the form of

5 $$\begin{array}{@{}rcl@{}} Q^{w}_{n}(\boldsymbol\beta_{\tau})=\sum\limits_{i=1}^{n}\sum\limits_{t=o_{i}}^{m}\rho_{\tau}\left(z_{it}^{w}-max\left\{{c_{i}}^{w},{\boldsymbol x_{it}^{w}}^{T}\boldsymbol \beta_{\tau}\right\}\right). \end{array} $$

Now the traditional censored quantile regression estimation algorithm ([4, 20]) can be straightly applied to minimize this objective function. Details related to estimation procedures, inference, and asymptotic properties of parameter estimators were discussed in Lee and Kong [10].

### A.2 Missing data process

If the missing early visit data arise from the MAR mechanism, estimation of probability of missing early visits is straightforward. To illustrate the missing data process, denote *R*_*it*_ as the missing status of response variable *z*_*it*_, i.e., *R*_*it*_=1 if *z*_*it*_ is observed and 0 otherwise. Then *R*_*ij*_=1 implies that $\phantom {\dot {i}\!}R_{ij'}=1$ for all *j*^′^>*j* given the monotone missing pattern. To indicate when the data collection is started, we define a random variable *O*_*i*_ as $O_{i}=1+(m-\sum _{t=1}^{m} R_{it})$. The probability of missing early visit $\pi _{io_{i}}$ from baseline to occasion *o*_*i*_−1 can be given by

$$\begin{array}{@{}rcl@{}} \pi_{io_{i}}&=& Pr(O_{i}=o_{i}|z_{io_{i}'}^{o}, \boldsymbol X_{i}, \boldsymbol \gamma) \\ &=&Pr\left(R_{i,1}, \cdots, R_{i,o_{i}-1}=0,R_{i,o_{i}}=1| z_{io_{i}'}^{o}, \boldsymbol X_{i}, \boldsymbol \gamma\right),  \end{array} $$

where $z_{io_{i}'}^{o}$ is the first observed *z* value after time *o*_*i*_ (i.e., *o*_*i*_<*oi*′≤*m*). When we define the probability of being observed at time *t* for the *i*-th subject as $\eta _{it}=Pr\left (R_{it}=1| R_{i,t+1}= \cdots = R_{im}=1, z_{it'}^{o}, \boldsymbol X_{i}, \boldsymbol \gamma \right)$, *t*<*t*^′^≤*m*, we can carry out the probability of missing early visits in terms of *η*_*it*_ as follows:

$${} {{\begin{aligned} \pi_{io_{i}}=& \prod\limits_{t=1}^{o_{i}-1} \left\{1- Pr\left(R_{it}=1| R_{i,t+1}= \cdots = R_{im}=1, z_{it'}^{o}, \boldsymbol X_{i}, \boldsymbol \gamma\right) \right\}  \\ & \times Pr\left(R_{io_{i}}=1| R_{i,o_{i+1}}= \cdots = R_{im}=1, z_{io_{i}'}^{o}, \boldsymbol X_{i}, \boldsymbol{\gamma}\right)^{I\{o_{i}\leq m\}} \\ =&\left (\prod_{t=1}^{o_{i}-1}(1-\eta_{it}) \right) (\eta_{io_{i}})^{I\{o_{i} \leq m \}},   \end{aligned}}}  $$

where ***γ*** is the parameter vector of the regression model for *η*_*it*_. Then appropriate regression models such as logistic regression model can be used to estimate *η*_*it*_, and then we can calculate $\pi _{io_{i}}$ based on the equation above.

### A.3 Multiple imputation process based on weighted censored quantile regression

Multiple imputation techniques [21] have been widely used for the general handling of missing data. However, the censoring and monotone missing mechanisms should be incorporated in the imputation models to deal with the complexity of missingness. Based on the weighted censored regression of quantiles that was introduced in the previous section, we propose the multiple imputation procedure to fill in the data that are left-censored or missing at early visits. The conditional censoring probability of *z*_*it*_, *ω*(***X***_*it*_)=*Pr*(*z*_*it*_<*c*|***X***_*it*_) can be estimated using a logistic regression model $logit[\omega (\boldsymbol X_{it}) ]=\boldsymbol X_{it}^{T}\boldsymbol \delta $, where *δ* is unknown parameter vector and then *v* is sampled from uniform distribution UNIF (0,*ω*(***X***_*it*_)) in order to impute the censored value *z*_*it*_ by its conditional quantile of ${z}_{it}^{*}=\boldsymbol X_{it}^{T} \hat {\boldsymbol \beta }_{v}$ which is estimated through fitting weighted censored quantile regression model where *z*_*it*_ is treated as the dependent variable as described in the previous section. We also draw *u* from UNIF(0,1) to fill in the missing value by ${z}_{it}^{*}=\boldsymbol X_{it}^{T} \hat {\boldsymbol {\beta }}_{u}$, that is the *u*-th conditional quantile of *z*_*it*_ given *X*_*it*_. Once the imputed datasets are generated, any analysis designed for the complete dataset can be applied to each of *M* imputed datasets. To obtain the parameter estimators of interest in the regression model $y_{it}= \alpha _{0}+\alpha _{1} z^{*}_{it}+\alpha _{2} w_{it}+\epsilon _{it}$, we define the combined MI estimator as $\hat {{\boldsymbol {\alpha }}}_{MI}=M^{-1}\sum _{k=1}^{M} \hat {{\boldsymbol {\alpha }}}_{k}$. Wang and Feng [28] discussed the asymptotic properties of their proposed multiple imputation procedure based on the conditional quantile function and suggested bootstrapping because the asymptotic variance of MI estimators takes complex forms and it is difficult to estimate directly. We adopted a bootstrap method by resampling the paired observations with replacement based on 500 bootstrap samples to obtain the standard errors for estimated parameters and *p*-values that were calculated by using the normality of estimated parameter $\hat {{\boldsymbol {\alpha }}}_{MI}$.

## Appendix B: Simulation study design

Suppose the subjects are to be observed at the same *m* time points. The latent longitudinal biomarker data are generated from the model

$$ \begin{aligned} z^{*}_{it}&=\beta_{0}+\beta_{1}x_{it}+\beta_{2}w_{it}+e_{it}-F^{-1}_{e_{it}},\\ i&=1,\cdots, n; \quad t=1,\cdots,m,  \end{aligned}  $$

where the covariates include a variable *x*_*it*_ with Poisson(20) distribution and *w*_*it*_ representing the *t*-th assessment time which is set equal to *t*. Given the covariates, random error vectors, ***e***_*i*_=(*e*_*i*1_,⋯,*e*_*im*_)^*T*^ for *i*=1,⋯,*n*, are assumed to be mutually independent and have conditional *τ*-th quantile equal to zero. Let us consider an error term $e_{it}-F^{-1}_{e_{it}}(\tau)$, where *F*(·) denotes the cumulative distribution function and $F^{-1}_{e_{it}}(\tau)$ is the *τ*-th quantile of *e*_*it*_ given *x*_*i*_ and *t*. We simulated the random variable *e*_*it*_ from each of the following distributions and calculated $F^{-1}_{e_{it}}(\tau)$ with *τ*=0.5 for each scenario.

1. Multivariate normal distribution (MVN); exchangeable covariance structure:
  ***e***_*i*_={*e*_*i*1_,⋯,*e*_*i*4_}^*T*^∼MVN(***0***,*σ*^2^***R***), where *σ*^2^=1 and correlation matrix ***R*** is exchangeable with *ρ*=0.3
$ \boldsymbol R=\left (\begin {array}{cccc} 1.0 & 0.3 & 0.3 & 0.3\\ & 1.0 & 0.3 & 0.3 \\ & & 1.0 & 0.3 \\ & & & 1.0 \\ \end {array}\right).$
2. Multivariate normal (MVN) distribution ; unstructured covariance:
  ***e***_*i*_={*e*_*i*1_,⋯,*e*_*i*4_}^*T*^∼MVN(***0***,*σ*^2^***R***), where *σ*^2^=1 and correlation matrix
$ \boldsymbol R=\left (\begin {array}{cccc} 1.00 & 0.75 & 0.44 & 0.54\\ & 1.00 & 0.37 & 0.46 \\ & & 1.00 & 0.08 \\ & & & 1.00 \\ \end {array}\right).$
3. Multivariate exponential distribution; exchangeable covariance:
  *e*_*it*_=*exp*(*ξ*_*it*_)−1 and ***ξ***_*i*_={*ξ*_*i*1_,⋯,*ξ*_*i*4_}^*T*^∼MVN(***0***,*σ*^2^***R***), where *σ*^2^=1 and ***R*** is exchangeable with *ρ*=0.3.
4. Multivariate exponential distribution; heteroscedastic covariance structure (i.e., covariance depends on a set of covariates ***X***_*i*_:
  *e*_*it*_=*exp*(*ξ*_*it*_)−1 and ***ξ***_*i*_={*ξ*_*i*1_,⋯,*ξ*_*i*4_}^*T*^∼MVN(***0***,1/(1+*x*_*i*1_)***R***), where ***R*** is exchangeable with *ρ*=0.3. Note that in this case, $F^{-1}_{e_{it}}(\tau)$ varies with *x*_*i*1_.

We set ***β***=(*β*_0_,*β*_1_,*β*_2_)^*T*^=(2.3,−0.25,−0.1)^*T*^, *m*=4 and overall censoring percentage *r*=10,15,20 or 30%. We chose the detection limit *c* as the (100 ×*r*)-th sample percentile of the simulated biomarker data $z^{*}_{it}$. Using latent variable $z^{*}_{it}$, we finally generated $y_{it}= \alpha _{0}+\alpha _{1} z^{*}_{it}+\alpha _{2} w_{it}+\epsilon _{it}$, (*α*_0_=5,*α*_1_=−4,*α*_2_=−6). As for the missing data process, the logistic regression model below was postulated,

6 $$\begin{array}{@{}rcl@{}} logit(\eta_{it}) = \gamma_{0}+\gamma_{1} z^{*}_{i,t+1} +\gamma_{2} x_{it},  \end{array} $$

where the parameter vector is ***α***=(*γ*_0_,*γ*_1_,*γ*_2_)^*T*^=(1,−8.5,0.5)^*T*^, *η*_*it*_ is the conditional probability of being observed at time *t*, and $z^{*}_{i,t+1}$ is the observed biomarker data at the time point *t*+1. Under this setting, we assumed the subjects with higher level of marker *z*^∗^ are more likely to have missing data.

## Abbreviations

AS: Ankylosing spondylitis
BASRI: Bath Ankylosing Spondylitis radiology index
CC: Complete case
CQR: Censored quantile regression
CRP: C-reactive protein
DL: Detection limit
ESR: Erythrocyte sedimentation rate
LOD: Limits of detection
MCMC: Markov Chain Monte Carlo
MI: Multiple imputation
mNY: Modified New York
mSASSS: Modified Stoke Ankylosing Spondylitis spine score
MSE: Mean squared error
MVE: Multivariate exponential
MVN: Multivariate normal
NSAIDs: Nonsteroidal Anti-Infammatory drugs
OMNI: Omniscient
PAH: Princess Alexandra Hospital in Brisbane, Australia
PSOAS: Prospective study of outcomes in Ankylosing spondylitis
RE: Relative efficiency
RR: Rate ratio
TNFi: Tumor Necrosis factors inhibitors
UCSF: University of California at San Francisco
UTH: University of Texas health science center at Houston
wCQR: Weighted CQR

## Footnotes

1 Participants from PAH have been enrolled since 2007.

2 **Study I-A**: 5-year funded study (enrolled from 2002–2006) for AS patients with disease symptom duration of > 20 years. Patients were initially enrolled for one visit but the protocol was amended to include the second follow up visit about 2 to 3 years after their initial enrollment; **Study I-B**: 2-year longitudinal study (enrolled from 2003–2006) for AS patients with disease symptom duration of < 20 years.
